# Automatic Cell Segmentation by Adaptive Thresholding (ACSAT) for Large-Scale Calcium Imaging Datasets

**DOI:** 10.1523/ENEURO.0056-18.2018

**Published:** 2018-09-13

**Authors:** Simon P. Shen, Hua-an Tseng, Kyle R. Hansen, Ruofan Wu, Howard J. Gritton, Jennie Si, Xue Han

**Affiliations:** 1 Department of Physics, Harvard University, Cambridge, MA 02138; 2 Biomedical Engineering Department, Boston University, Boston, MA 02215; 3 School of Electrical, Computer and Energy Engineering, Arizona State University, Tempe, AZ 85287

**Keywords:** GCaMP6, genetically encoded calcium sensors, in vivo imaging, adaptive thresholding, ROI segmentation, automated image analysis, wide-field imaging, two-photon imaging, neural network

## Abstract

Advances in calcium imaging have made it possible to record from an increasingly larger number of neurons simultaneously. Neuroscientists can now routinely image hundreds to thousands of individual neurons. An emerging technical challenge that parallels the advancement in imaging a large number of individual neurons is the processing of correspondingly large datasets. One important step is the identification of individual neurons. Traditional methods rely mainly on manual or semimanual inspection, which cannot be scaled for processing large datasets. To address this challenge, we focused on developing an automated segmentation method, which we refer to as automated cell segmentation by adaptive thresholding (ACSAT). ACSAT works with a time-collapsed image and includes an iterative procedure that automatically calculates global and local threshold values during successive iterations based on the distribution of image pixel intensities. Thus, the algorithm is capable of handling variations in morphological details and in fluorescence intensities in different calcium imaging datasets. In this paper, we demonstrate the utility of ACSAT by testing it on 500 simulated datasets, two wide-field hippocampus datasets, a wide-field striatum dataset, a wide-field cell culture dataset, and a two-photon hippocampus dataset. For the simulated datasets with truth, ACSAT achieved >80% recall and precision when the signal-to-noise ratio was no less than ∼24 dB.

## Significance Statement

ACSAT aims at automatically segmenting cells in large-scale calcium imaging datasets. It is based on adaptive thresholding at both global and local levels and iteratively identifies individual neurons in a time-collapsed image. It is designed to address a variety of datasets, potentially involving variations in cell morphology and fluorescence intensity between different datasets. We demonstrate the effectiveness of ACSAT by testing it under a variety of conditions. For the simulated datasets with truth, ACSAT achieved recall and precision rates >80% when the signal-to-noise ratio was no less than ∼24 dB. For the datasets from mouse hippocampus and striatum, ACSAT captured ∼80% of human-identified ROIs and even detected some low-intensity neurons that were initially undetected by human referees.

## Introduction

The ability to record from a large population of single neurons during behavior greatly facilitates the investigation of the contribution of individual neurons to neuronal network dynamics. Extracellular single-unit recording has traditionally been a method of choice in neurophysiological analyses of single neurons in the brain. Recent improvements, such as the new generation of genetically encoded calcium sensors GCaMP6 (Chen et al., 2013, Sun et al., 2013), have made it possible to observe hundreds to thousands of individual neurons simultaneously (Ohki et al., 2005; Andermann et al., 2010; Huber et al., 2012; Ziv et al., 2013; Issa et al., 2014; Mohammed et al., 2016). Though indirect, these calcium indicators have been sensitive enough to monitor neuronal activity with high spatiotemporal precision in behaving animals, allowing researchers to examine the activity of populations of a specific cell type (Hofer et al., 2011; Wachowiak et al., 2013; Pinto and Dan, 2015; Allen et al., 2017) or the same cell over an extended period of time (Poort et al., 2015).

As the performance of genetically encoded calcium indicators has improved, wide-field microscopy has become feasible for recording the activity of a large population of neurons over an extended anatomical area (Lütcke et al., 2013; Wilt et al., 2013). Although lacking the spatial subcellular resolution of a multiphoton microscope, wide-field microscopes can operate at a higher speed, allowing simultaneous recording of increasingly larger populations (Ghosh et al., 2011; Ziv et al., 2013; Kim et al., 2016; Mohammed et al., 2016). Advanced microfabrication techniques further miniaturized the wide-field microscope to a microendoscope capable of monitoring neural activity in freely-moving animals (Ghosh et al., 2011; Ziv et al., 2013).

An emerging technical challenge that parallels advances in calcium imaging is the processing of large datasets (Hamel et al., 2015). During data analysis, an important step is to identify regions of interest (ROIs) corresponding to individual neurons. As data grows rapidly both spatially and temporally, the traditional labor-intensive approach of manual inspection has to be automated. Principal component analysis (PCA) and independent component analysis (ICA) methods are natural and frequently used candidates for automating ROI identification (Mukamel et al., 2009). However, if its assumption of statistical independence between neurons is violated, which is often the case in real neural recordings, then the method relies on user selection of parameters for spatial segmentation.

Threshold-based methods represent a promising and intuitive alternative for automatic ROI identification. However, several challenges need to be overcome, including variability in recording conditions or fluorescence signal strength across structures, recording subjects, and the imaging field. For example, one of the most referenced thresholding methods, Otsu’s method, which automatically selects the optimal threshold value that minimizes the intraclass variance among ROI pixels and among background pixels, would only successfully segment some of the highest-intensity ROIs (Otsu, 1979; Sezgin and Sankur, 2004). Additionally, the multiclass Otsu’s method is limited because uneven lighting may result in separate background classes. A waterfall-thresholding approach addresses uneven lighting by iterative thresholding to capture all intensity peaks, but its selection of a threshold value is ad hoc, making it dataset-dependent and user-dependent (Mellen and Tuong, 2009). A feedback loop–based approach for segmenting bacteria cells optimizes the threshold value from the distribution of pixel intensities, but its assumption that the total ROI area remains constant over time does not hold for calcium-imaging datasets because neurons change in brightness (Shen et al., 2015). A recent machine learning–based algorithm uses image gradients and pixel traces to optimize threshold values, but it still requires a user’s subjective input in selecting a background removal factor based on each dataset (Fantuzzo et al., 2017). Other approaches based on edge detection have trouble due to weak fluorescence signal strength in comparison with the background pixels (Sadeghian et al., 2009). Generally, most segmentation methods require a high level of tuning to each individual dataset.

To overcome these challenges of diverse imaging datasets, we introduce a new automated cell segmentation by adaptive thresholding (ACSAT) algorithm. ACSAT dynamically and automatically determines global and local threshold values based on the distribution of pixel intensities within a time-collapsed image of a recorded image sequence. We demonstrate the utility of ACSAT on simulated datasets, cell culture datasets, and *in vivo* wide-field and two-photon datasets. For the simulated datasets with truth, ACSAT achieved >80% recall and precision when the signal-to-noise ratio was no less than ∼24 dB. ACSAT also captured ∼80% of human-identified ROIs in datasets from mouse hippocampus and striatum and was even able to detect low-intensity neurons that were initially undetected by human referees.

## Materials and Methods

### Wide-field hippocampus and striatum datasets

All animal procedures were approved by [Boston University] Institutional Animal Care and Use Committee. Female C57BL/6 mice (8–12 weeks old, Taconic) were first injected with 250 nL AAV9-Syn-GCaMP6.WPRE.SV40 virus (titer: ∼6e12 GC/mL, University of Pennsylvania Vector Core). AAV was delivered either into the dorsal CA1 (AP: –2, ML: 1.4, DV: –1.6), or into the dorsal striatum (AP: 0.5, ML: 1.8, DV: –1.6) regions. Injections were performed with a 10-μL syringe (World Precision Instruments) coupled with a 33-gauge needle (NF33BL, World Precision Instruments) at a speed of 40 nL/min, controlled by a microsyringe pump (UltraMicroPump 3-4, World Precision Instruments). Upon complete recovery, a custom imaging chamber with glass coverslip was surgically implanted on top of the viral injection site by removing the overlying cortical tissue. The imaging chamber was assembled by fitting a circular coverslip (size 0; OD: 3 mm) to a stainless steel cannula (OD: 3.17 mm, ID: 2.36 mm) using a UV-curable optical adhesive (Norland Products). During surgery, a custom aluminum headplate was also attached to the skull, which allowed head fixation during the imaging session.

Imaging data were acquired with a custom wide-field microscope coupled with a scientific CMOS camera (ORCA-Flash 4.0, C11440-42U, Hamamatsu), controlled by the commercial software package HCImageLive (Hamamatsu). The wide-field microscope consisted of a Leica N Plan 10× 0.25 PH1 objective lens, an excitation filter (HQ 470/50), a dichroic mirror (FF506-Di02), an emission filter (FF01-536/40), a commercial SLR lens as the tube lens (Nikon Zoom-NIKKOR 80–200 mm f/4 AI-s), and a 5W LED (LZ1-00B200, 460 nm; LedEngin). Data acquisition was performed at 20 Hz, at a resolution of 1024 × 1024 pixels, with 16 bits per pixel, for ∼10–20 min. With 10× objective lens, the microscope provided a field of view of 1.343 × 1.343 mm^2^ (1.312 × 1.312 μm^2^/pixel) of brain tissue. Imaging data were streamed from the camera to RAM of a custom computer (dual Intel Xeon processors, 128 GB RAM, and a GeForce GTX Titan video card) to ensure temporal precision. After each imaging session, data were moved from RAM to hard drive and saved in multipage tagged image file format.

Two hippocampus datasets (A and B) were collected from two mice [dataset A was previously reported by Mohammed et al. (2016)]. The mice were trained to perform a trace conditioning task known to involve hippocampal neural activity (Solomon et al., 1986; Moyer et al., 1990; Tseng et al., 2004; Sakamoto et al., 2005). In this task, the animal was trained to associate a conditioned stimulus (a 350-ms-long tone) with an unconditioned stimulus (a gentle 100-ms air puff to one eye). There was a 250-ms trace interval between two stimuli. During each recording session, the animal was head-fixed and performed 40 trials with a randomized 31–36-s intertrial interval. The hippocampus datasets (1024 × 1024 pixels/frame, 2047 frames, ∼100 s, ∼4 GB size) analyzed in this study were part of larger recording sessions (∼50 GB size).

The striatum dataset was collected from a head-fixed animal running on a spherical treadmill system. The treadmill system consisted of a Styrofoam ball floated by air pressure in a 3D-printed bowl designed as described in Dombeck et al. (2007) that allowed the animal to move its limbs freely while head-fixed. The mouse was first handled for several days before being head-fixed to the spherical treadmill. Habituation to running on the spherical treadmill while head-fixed occurred over 3–4 days/week at the same time of day as subsequent recording sessions (8–12 h after lights-on), for several weeks. Single imaging sessions took ∼25 min. Sampling occurred at ∼20 Hz, and exposure time was fixed at 20 ms. The striatum dataset (∼100 s, ∼4 GB size) contains 2047 frames with 1024 × 1024 pixels per frame and was also part of a larger dataset (∼25 GB size).

Two human referees manually identified ROIs in the hippocampus dataset A and in the striatum dataset to create a set of human-generated ROIs for comparison with ACSAT’s segmentation results. This manual selection was done by viewing the image sequence and segmenting ROIs that had fluorescence traces compatible with neuronal dynamics and/or by selecting ROIs from a composite image created from the video sequence and confirming that fluorescence traces were compatible with neuronal dynamics.

### Wide-field cell culture dataset

The primary neuron cell dataset was collected from a 10-day-old neuron culture, infected with AAV9-Syn-GCaMP6.WPRE.SV40 virus. Seven days after infection, neurons were imaged at 20 Hz for 60 s. The primary neuron culture dataset contains 1201 frames, 1024 × 1024 pixels per frame, recorded with the same imaging setup as for the hippocampus and striatum datasets described above.

### Two-photon dataset

The two-photon dataset was downloaded from the Neurofinder website (http://neurofinder.codeneuro.org/, 03.00). GCaMP6f was used as the indicator. The dataset contains 2250 frames with 498 × 490 pixels per frame with resolution 0.588 × 0.588 μm^2^/pixel.

### Signal-to-noise ratio (SNR) calculation

We calculated the SNR in decibels (dB) as

$$\text{SNR}=20\times \log\frac{\mu_{\text{ROI}}}{\sigma_{\text{background}}}.$$

For the simulated datasets, $\mu_{\text{ROI}}$ is the mean intensity value of all pixels belonging to all ROIs in the time-collapsed image $I_{0}$, and similarly, $\sigma_{\text{background}}$ is the standard deviation of background pixel intensity values, i.e., all pixels that do not belong to an ROI. For the hippocampus dataset A and the striatum dataset, $\mu_{\text{ROI}}$ is the maximum-intensity value of an ROI trace, and $\sigma_{\text{background}}$ is the standard deviation of the background trace. The ROI trace value at each time point is the averaged intensity values of all pixels belonging to that ROI, and similarly for the background trace, which uses all pixels not belonging to any ROI. Note that the SNR for the simulated datasets describes the whole time-collapsed image $I_{0}$, whereas the SNRs for the hippocampus and striatum datasets describe an individual ROI.

### Simulated datasets

**Figure 1.**
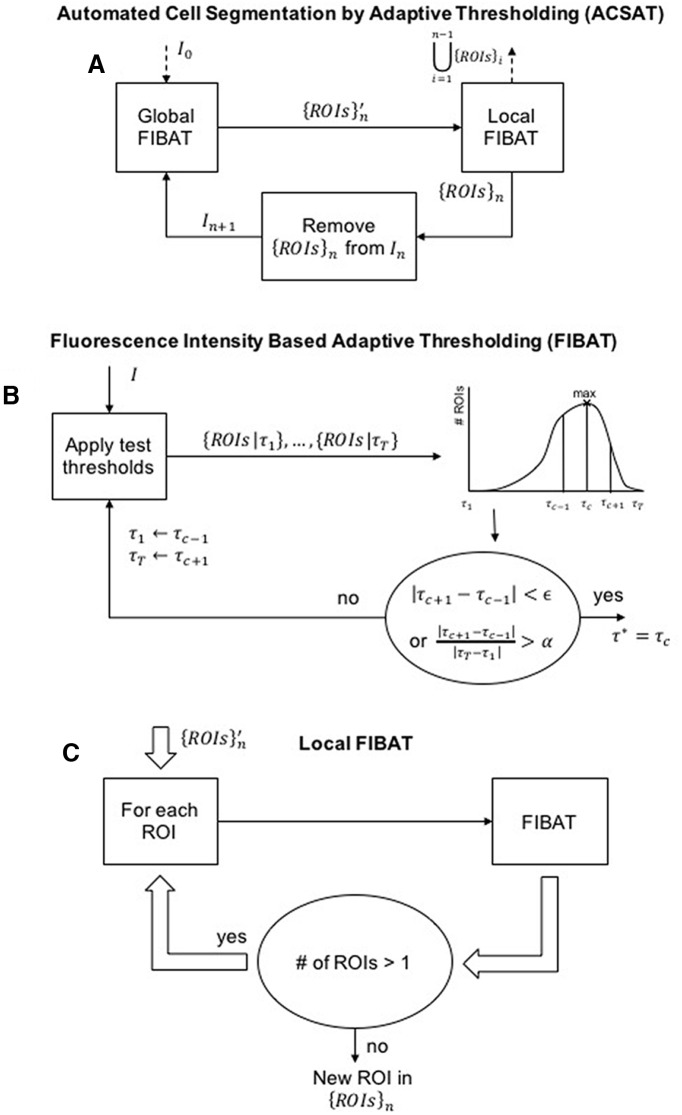
Flowchart of the ACSAT algorithm. ***A***, The input is the time-collapsed image $I_{0}$, and the output is a collection of automatically segmented ROIs. In each iteration, the Global FIBAT step identifies potential ROIs ${\left\{ROIs\right\}}_{n}^{'}$ by applying FIBAT, described in ***B***, to the entire image $I_{n}$; and the Local FIBAT step, described in ***C***, splits overlapping ROIs. ***B***, Flowchart of the FIBAT algorithm. The input image is segmented using each of the test threshold values $\tau_{1},\tau_{2},\ldots ,\tau_{T}$. The search range for the optimal threshold value ($\tau_{1},\tau_{T}$) is iteratively narrowed to contain the test threshold value which results in the maximum number of ROIs. ***C***, Local FIBAT procedure. FIBAT, described in ***B***, is recursively applied to each potential ROI until the resulting ROIs can no longer be separated by FIBAT.

**Figure 2.**
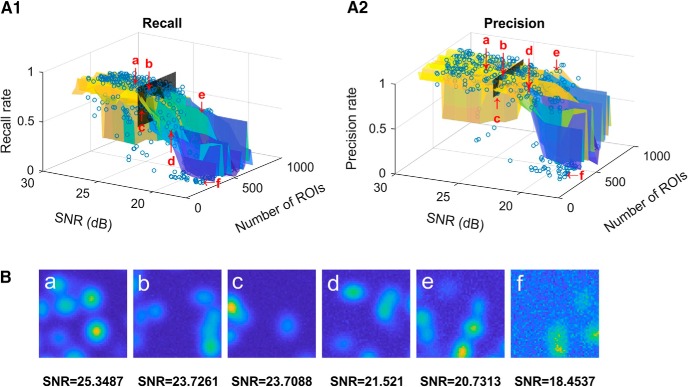
ACSAT performance on simulated datasets. ***A1***,***A2***, Recall (***A1***) and precision (***A2***) are plotted as a function of SNR and number of ROIs. Each dot corresponds to the ACSAT result for one of the 500 simulated datasets. A surface was fitted to these dots for visualization. The black vertical plane corresponds to the SNR of the hippocampus A dataset. ***B***, Six examples of simulated time-collapsed images, labeled a–f, correspond to the dots labeled a–f in ***A1*** and ***A2***.

We tested ACSAT’s segmentation performance on 500 simulated datasets with varying SNRs (between ∼19 and ∼29 dB) and numbers of ROIs (between 300 and 700). Fig. 2*B* shows some examples of the simulated time-collapsed image, i.e., the input image $I_{0}$ to ACSAT in Fig. 1*A*. The simulation gives us the true locations of all ROIs so that we can accurately assess ACSAT’s segmentation performance.

Our simulated datasets were obtained by a procedure adapted from Zhou et al. (2018). We used the model $I_{0}=E+AC$ to generate the simulated datasets, where *E* represents noise, *A* represents the shapes of each ROI, and *C* adjusts each ROI’s intensity to simulate uneven lighting.

The pixel noise values in *E* were randomly sampled from the background pixel values in the time-collapsed image for the hippocampus dataset. This noise is unlikely to be Gaussian, because the time-collapsing procedure subtracts the mean value from the maximum value of each pixel such that the time-collapsed image is biased toward higher pixel values.

The centroid location of each ROI represented in *A* was randomly selected with weights *C* ^2^. The pixel values comprising the body of each ROI was modeled deterministically by the bivariate Gaussian probability density function, with widths randomly selected according to Zhou et al. (2018).

The image *C* is also used to amplify each ROI’s pixel values to reflect uneven lighting conditions across the imaging field. *C* was generated by applying heavy Gaussian filtering to the time-collapsed image of the hippocampus dataset until no individual ROIs are detectable.

### ACSAT overview

Fluorescence imaging data obtained in the form of image sequences is processed offline using a custom Matlab algorithm. Image sequences were first motion-corrected as described in Mohammed et al. (2016) to remove micromotion of the imaged area caused by breathing and other movements of the animal. ACSAT (Fig. 1*A*) is then applied to a time-collapsed image that represents the image sequences, to automatically identify individual neurons as ROIs.

The input image sequence is first loaded into Matlab as a 3D matrix (height × width × time) and then time-collapsed to produce a representative two-dimensional image (height × width, $I_{0}$ in Fig. 1*A*), where each pixel in $I_{0}$ is represented by the maximum-intensity value of that pixel across the entire image sequence with its mean value removed. This time-collapsed image $I_{0}$ is then used for the rest of the algorithm. Pixels with low-intensity values would correspond to static background, whereas pixels with high-intensity values would correspond to neurons with GCaMP6 expression. In general, neurons with GCaMP6 expression appear in $I_{0}$ as a cluster of adjacent pixels with high-intensity values and with size similar to that of a neuron. Meanwhile, it is improbable for random background noise to generate clusters with similar properties. Thus, the time-collapsed image $I_{0}$ is expected to contain sufficient information to separate neurons from the background.

Next, ACSAT iteratively generates ROIs ${\left\{ROIs\right\}}_{n}$ from the time-collapsed image $I_{n}$ for iterations n=1,2,…, starting with $I_{1}=I_{0}$. Before each subsequent iteration, $I_{n}$ is generated by cumulatively clearing previously segmented ROIs, ${\left\{ROIs\right\}}_{n-1}$, from $I_{n-1}$ by setting each ROI’s pixels in $I_{n-1}$ to blank values of 0 and dilating the cleared area. As described later, each iteration consists of both adaptive thresholding at the global level (Global FIBAT in Fig. 1*A*), using the automatically selected threshold value $\tau_{n}^{*}$ (Fig. 1*B*), and adaptive thresholding at the local level (Local FIBAT in Fig. 1*A*). When the change in global threshold value $\left|\tau_{n+1}^{*}-\tau_{n}^{*}\right|$ is insignificant, further iterations are likely to contribute more false positives than true positives. Thus, the ACSAT algorithm terminates at iteration n if

$$\frac{\left|\tau_{n+1}^{*}-\tau_{n}^{*}\right|}{\tau_{1}^{*}}<\delta ,$$

where $\tau_{1}^{*}$ acts as a normalizing factor. Accordingly, the final output of ACSAT is the union of the segmented ROIs from each iteration, ${\left\{ROIs\right\}}_{1}\cup \cdots \cup {\left\{ROIs\right\}}_{n}$.

### Global and local adaptive thresholding in ACSAT

Each iteration n of ACSAT contributes a set of newly segmented ROIs ${\left\{ROIs\right\}}_{n}$ from $I_{n}$ by applying our fluorescence intensity based adaptive thresholding (FIBAT) algorithm, at the global and local levels (Global/Local FIBAT in Fig. 1*A*). Briefly, FIBAT (Fig. 1*B*) takes an inputted image I and outputs the optimal threshold value $\tau^{*}$ which results in optimally segmented ROIs $\left\{ROIs|\tau^{*}\right\}$.

Global adaptive thresholding is the first step in the *n*th iteration of ACSAT (Fig. 1*A*). This step applies FIBAT directly to the whole image $\left(I_{n}\to I\right)$ to identify potential ROIs $\left(\left\{ROIs|\tau^{*}\right\}\to {\left\{ROIs\right\}}_{n}^{\prime }\right)$.

These potential ROIs ${\left\{ROIs\right\}}_{n}^{\prime }$ may include groups of adjacent neurons or overlapping neurons because neurons located above and below the focal plane could be captured in the same frame during wide-field imaging. Such overlap, however, is unlikely to occur in two-photon datasets or in cell culture datasets. The local adaptive thresholding step (Fig. 1*C*) recursively separates any potentially overlapping ROIs within ${\left\{ROIs\right\}}_{n}^{\prime }$ to output ${\left\{ROIs\right\}}_{n}$. Specifically, each ROI in ${\left\{ROIs\right\}}_{n}^{\prime }$ is individually dilated and then inputted to the local FIBAT (ROI→I) in Fig. 1*B* to obtain a set of separated ROIs $\left\{ROIs|\tau^{*}\right\}$. If any outputted set $\left\{ROIs|\tau^{*}\right\}$ contains more than one separated ROI, then each ROI in the set $\left\{ROIs|\tau^{*}\right\}$ is further separated using the same procedure, thus forming a recursive loop. Otherwise, if any outputted set $\left\{ROIs|\tau^{*}\right\}$ contains only one ROI, then the recursion terminates. The final output of the local thresholding step ${\left\{ROIs\right\}}_{n}$ is the union of all such sets containing one ROI that cannot be further separated.

### FIBAT

As described, FIBAT (Fig. 1*B*) is used in both the global and local adaptive thresholding steps of each iteration of ACSAT to identify potential ROIs in the time-collapsed image $I=I_{n}$ or to separate potentially overlapping neurons within *I* which is an element of ${\left\{ROIs\right\}}_{n}^{\prime }$, respectively. In either case, an optimal pixel intensity threshold value $\tau^{*}$ separates ROIs from the background. FIBAT selects $\tau^{*}$ by searching for the threshold value that maximizes the number of resulting ROIs that are larger in area than $A_{\min}$ and smaller in area than $A_{\max}$.

The search is performed recursively over a pixel intensity range $\left(\tau_{1},\tau_{T}\right)$, where initially $\tau_{1}$ is the minimum pixel intensity value in *I* and $\tau_{T}$ is the maximum pixel intensity value in *I*. From this search range, *T* test threshold values $\tau_{1},\tau_{2},\ldots ,\tau_{T-1},\tau_{T}$ are uniformly selected. A larger *T* will decrease the probability of skipping the optimal threshold value, but it will result in more computation time that may not be necessary. Because the threshold value is refined by a recursive process until it reaches the optimal value that produces the maximum number of ROIs, the value of *T* should have little to no effect on ACSAT’s segmentation results. We chose T=12. Each of these test threshold values $\tau_{1},\ldots ,\tau_{T}$ is applied to the image *I* by assigning each pixel a 1 (a true calcium event) if its value is greater than the threshold or a 0 (a false calcium event) otherwise. Morphological operations are then performed to refine the thresholded images. Specifically, these operations fill in holes (0s surrounded by 1s) and remove spur pixels that may be due to noise. The operations also break H-connected ROIs before splitting overlapping cells. ROIs are finally collected with 8-connectivity (Matlab function bwlabel or bwconncomp) to generate a set of segmented ROIs for each test threshold value: $\left\{ROIs|\tau_{1}\right\},\ldots ,\left\{ROIs|\tau_{T}\right\}$.

Since ROIs represent real neurons that are roughly spherical in shape and are ∼5–20 μm in diameter, some realistic criteria can be used to eliminate false ROIs that are not possibly actual neurons. Accordingly, FIBAT removes ROIs from $\left\{ROIs|\tau_{1}\right\},\ldots ,\left\{ROIs|\tau_{T}\right\}$ if their centroid is outside the ROI, or if their area is less than $A_{\min}$ or greater than $A_{\max}$, or if their solidity (i.e., the area ratio between the convex hull of a ROI and the ROI itself) is greater than approximately the golden ratio.

The next search range is selected based on the results of the test thresholds. A relationship of the test threshold values $\tau_{1},\ldots ,\tau_{T}$ versus the numbers of resulting ROIs $\left|\left\{ROIs|\tau_{1}\right\}\right|,\ldots ,\left|\left\{ROIs|\tau_{T}\right\}\right|$ can be generated (Fig. 1*B*). If the test threshold value $\tau_{c}$ resulted in the most ROIs, i.e., $c=\arg\max_{c}\left|\left\{ROIs|\tau_{c}\right\}\right|$, then the next search range is set to $\left(\tau_{\max\left\{1,c-1\right\}},\tau_{\min\left\{T,c+1\right\}}\right)$ to include $\tau_{c}$ inside the search range. If more than one test threshold value $\tau_{c_{1}},\tau_{c_{2}},\ldots$ resulted in the same maximum number of ROIs, then the next search range is similarly set to $\left(\tau_{\max\left\{1,\min\left\{c_{1},c_{2},\ldots \right\}-1\right\}},\tau_{\min\left\{T,\max\left\{c_{1},c_{2},\ldots \right\}+1\right\}}\right)$ to contain all $\tau_{c_{1}},\tau_{c_{2}},\ldots$. This search is terminated when further refinement of the search range produces little improvement in the number of detected ROIs: either the new search range $\left|\tau_{c+1}-\tau_{c-1}\right|$ is less than ε or the new range overlaps the previous range by at least α. We chose α=90% and ε= the smallest nonzero intensity difference between every pair of adjacent pixels in whole image *I*. As such, ε is determined automatically and does not require user input. On termination, the optimal threshold value is set to $\tau^{*}=\frac{1}{2}\left(\min\left\{\tau_{c_{1}},\tau_{c_{2}},\ldots \right\}+\max\left\{\tau_{c_{1}},\tau_{c_{2}},\ldots \right\}\right)$, and the segmented ROIs $\left\{ROIs|\tau^{*}\right\}$ includes ROIs whose area exceeds $A_{\max}$.

### Code accessibility

The code/software described in the paper is freely available online at https://github.com/sshen8/acsat. The code is available in Extended Data 1.

10.1523/ENEURO.0056-18.2018.ed1 Extended Data 1 ZIP file contains 11 Matlab files which comprise the ACSAT algorithm. Download Extended Data 1, ZIP file.

## Results

We tested ACSAT on 500 simulated datasets, two wide-field hippocampus datasets, a wide-field striatum dataset, a wide-field cell culture dataset, and a two-photon hippocampus dataset. The simulated datasets with known ground truth allowed us to accurately assess the segmentation performance of ACSAT in different conditions of SNR and number of ROIs. For the hippocampus dataset A and the striatum dataset, in which the ground truth is unknown, we used human-generated ROIs as a reference. For the cell culture dataset, hippocampus dataset B, and two-photon dataset, we provide the ACSAT segmented ROIs that can be inspected and interpreted by users.

### ACSAT performance on simulated datasets with various SNRs and numbers of ROIs

To evaluate the performance of ACSAT, we simulated 500 time-collapsed images $I_{0}$ with various numbers of ROIs (between 300 and 700) at random locations and different SNRs (between ∼19 and ∼29 dB). The exact locations of ROIs are known and served as the ground truth to provide an accurate evaluation of the performance of ACSAT. For all 500 datasets, we used the parameters δ=10%, Amin=50 px≈86 µm2 and Amax=300 px≈516 µm2 for the global adaptive thresholding step, and Amin=20 px≈34 µm2 and $A_{\max}=\infty$ for the local adaptive thresholding step because ROIs tend to shrink in size after repeatedly applying FIBAT.

The recall and precision results for each of these simulated datasets are shown as dots in Fig. 2*A1* and Fig. 2*A2*, respectively. Fig. 2*B* shows examples of the simulated time-collapsed images, and each example corresponds to a dot in Fig. 2*A1* and Fig. 2*A2*. At SNR greater than ∼24 dB, ACSAT shows a stable performance with generally higher than 80% recall. The precision rate remains stable at generally higher than 80% when SNR is greater than ∼21 dB. However, the performance of ACSAT falls when SNR is below ∼20 dB.

### ACSAT performance on hippocampus dataset A and striatum dataset

**Figure 3.**
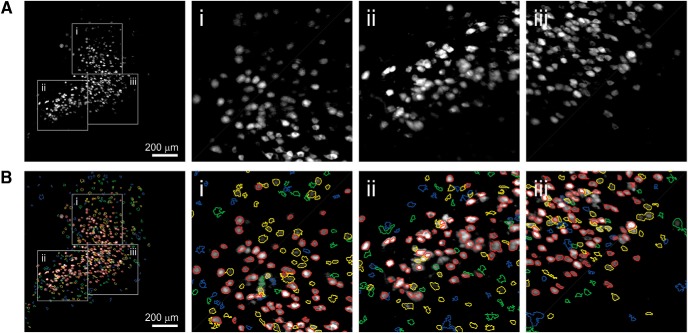
Hippocampus dataset A and ROIs identified by ACSAT. ***A***, The time-collapsed image of hippocampus dataset A and zoom-in images (***Ai***, ***Aii***, and ***Aiii***, corresponding to the gray boxes). ***B***, ACSAT-determined ROIs from multiple iterations overlying on the time-collapsed image (red, yellow, green, and blue outline corresponds to iteration 1, 2, 3, and 4, respectively). The fourth iteration (blue) is shown for comparison although ACSAT terminated at iteration 3 (red, yellow, and green).

**Figure 4.**
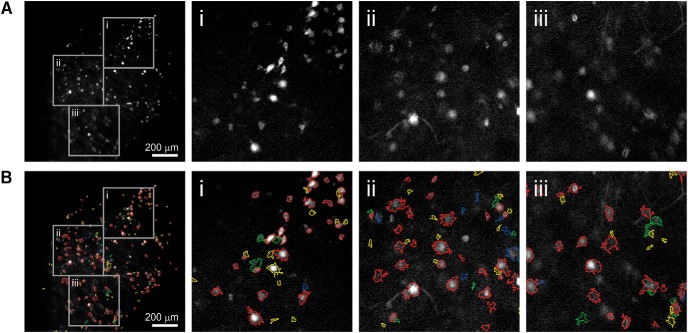
Striatum dataset and ROIs identified by ACSAT. ***A***, The time-collapsed image of striatum dataset and zoom-in images (***Ai***, ***Aii***, and ***Aiii***, corresponding to the gray boxes). ***B***, ACSAT-determined ROIs from multiple iterations overlying on the time-collapsed image (red, yellow, green, and blue outline corresponds to iteration 1, 2, 3, and 4, respectively). The second (yellow), third (green), and fourth (blue) iterations are shown for comparison although ACSAT terminated at iteration 1 (red).

We used ACSAT (Fig. 1*A*) to automatically segment ROIs from a hippocampus wide-field imaging dataset and a striatum wide-field imaging dataset. Before the application of the ACSAT, the image sequences were time-collapsed as shown in Figs. 3 and 4 (top rows) for the hippocampus A and the striatum datasets, respectively. These time-collapsed images show high-intensity areas resembling neural morphology. The final segmented ROIs outputted by ACSAT are illustrated in Figs. 3 and 4 (bottom row), respectively.

For both datasets, we initiated ACSAT using the same parameters as for the simulated datasets (δ=10%, Amin=50 px≈86 µm2 and Amax=300 px≈516 µm2 for the global adaptive thresholding step, and Amin=20 px≈34 µm2 and $A_{\max}=\infty$ for the local adaptive thresholding step). To obtain the results as shown in Figs. 3 and 4, it took approximately 1 min per iteration on a Xeon E5-1650 v4 at 3.6 GHz with 128 GB DDR4 RAM, but it used <30 MB RAM. As such, the RAM size had little effect on the speed.

#### ACSAT performance compared to human-generated ROIs

To assess the performance of the ACSAT algorithm, we compared the ACSAT segmentation results with ROIs generated by human inspection (human-generated ROIs). This set of human-generated ROIs contained 423 ROIs for the hippocampus dataset A and 91 ROIs for the striatum dataset. We first compared the ACSAT-generated ROIs for the hippocampus A and striatum datasets with the ROIs in the human-generated ROIs. We consider a pair of ROIs to correspond to the same neuron if they had centroids that were <50 px≈65.6 µm apart and had a mutual overlap >60%. We calculated the mutual overlap as the average of the percentages of the overlapping area against the areas of both ROIs. When there were multiple ROIs sharing overlapping areas, we selected the pair with highest mutual overlap as the matched ROIs.

For the hippocampus dataset A, ACSAT identified 445 ROIs after three iterations. Among these 445 ROIs, 317 ROIs were matched in the human-generated ROIs (Match), and 128 ROIs were not in the human-generated ROIs (ACSAT-only). Additionally, 106 ROIs in human-generated ROIs were not identified by ACSAT (Human-only). This result gave us a precision rate of 71.2% (317 out of 445) and a recall rate of 74.9% (317 out of 423). For the striatum dataset, ACSAT was terminated after one iteration and identified a total of 135 ROIs: 69 Match ROIs, 66 ACSAT-only ROIs, and 22 Human-only ROIs (precision rate: 51.1%, recall rate: 75.8%).

**Figure 5.**
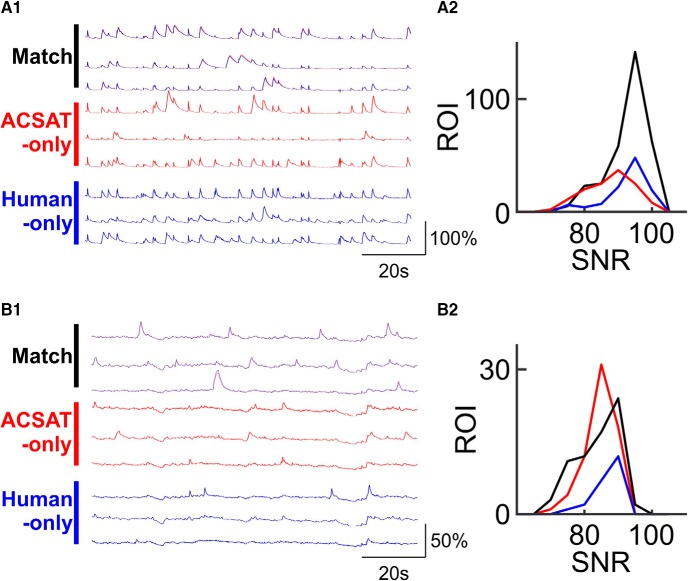
Fluorescence traces and SNRs. ***A1***, Representative fluorescence traces from the hippocampus dataset A for ROIs identified by both ACSAT and human referees (Match), ROIs identified only by ACSAT, and ROIs identified only by human referees (Human). ***A2***, Histogram of SNR for Match, ACSAT, and human ROIs from the hippocampus dataset A. ***B1***, Representative fluorescence traces from the striatum dataset. ***B2***, Histogram of SNR for the striatum dataset.

We further examined the fluorescence traces of ROIs from the ACSAT-only, Human-only, and Match groups. Representative traces are shown in Fig. 5*A1*,*B1*, respectively, for the hippocampus A and striatum datasets, and all traces are available in Extended Data 2. The value of each ROI fluorescence trace at each time point is the average intensity value of all pixels belonging to that ROI. In Fig. 5*A1*,*B1*, each trace is normalized by subtracting the mean value of that trace over time and then dividing the difference by that mean value. We calculated the SNR for every ROI in each group. In both the hippocampus A and striatum datasets, the Match ROIs exhibit a broad range of SNR, indicating that both ACSAT and humans are capable of identifying ROIs with various intensities in the time-collapsed image (Fig. 5*A2*,*B2*).

We further examined the individual ROIs identified by ACSAT that were not identified by humans. This secondary manual inspection found that some of the ACSAT-only ROIs were actually true neurons (i.e., with fluorescence traces compatible with neuronal dynamics) that were missed in the initial human-generated ROIs because of human error. This means that ACSAT was able to segment ROIs that were difficult to identify by human experts. Specifically, for the hippocampus A dataset, 70 (54.7%) out of 128 ROIs initially labeled as ACSAT-only were later determined to be actual neurons, and for the striatum dataset, 31 (47%) ROIs were true neurons. After correction, of the total 445 ACSAT ROIs from the hippocampus dataset A, 387 segmented ROIs corresponded to true neurons (Match), and 58 segmented ROIs did not correspond to true neurons determined by human inspection (ACSAT-only). Additionally, 106 true ROIs were not segmented (Human-only). This corresponds to a precision rate of 87% and a recall rate of 78.5%. Similarly, for the striatum dataset, which resulted in 135 ACSAT ROIs, there were 100 Match ROIs, 35 ACSAT-only ROIs, and 22 Human-only ROIs after correction. This corresponds to a precision rate of 74.1% and a recall rate of 82%. Although neurons in the hippocampus and striatum have different morphology and fluorescence intensity, ACSAT was consistently effective for both datasets, and it was able to detect low-intensity neurons that were initially undetected by human referees. As such, our results demonstrate the robustness and effectiveness of the algorithm.

**Figure 6.**
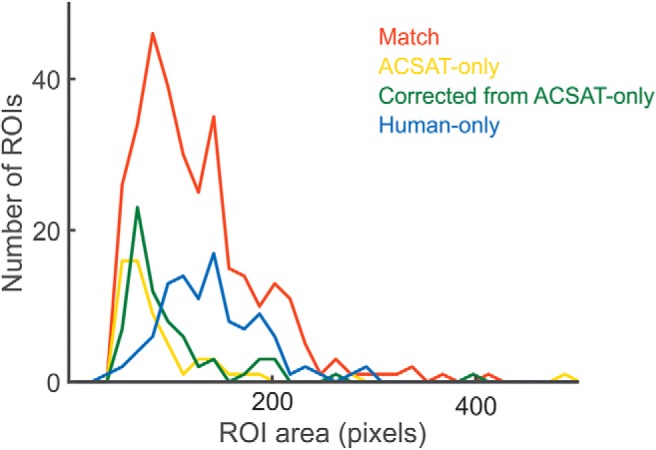
Distribution of ROI size for hippocampus dataset A. ROIs identified by ACSAT and human (red) with various size. The ACSAT-only ROIs (yellow) and those missed by human experts (green) tend to have small areas, while the areas of human-only ROIs (blue) appear slightly larger.

The result from the hippocampus dataset A shows that ACSAT successfully identified true ROIs of diverse sizes (Fig. 6, red). In general, the false-positive ROIs had relatively smaller areas (Fig. 6, yellow), similar to the ROIs missed by human referees (Fig. 6, green). This indicates that ACSAT is more likely to recognize intensity changes in small areas, thereby outperforming human referees under such challenging detection conditions. Additionally, ACSAT missed a small portion of true ROIs, which shares similar sizes with those identified (Fig. 6, blue).

#### Number of iterations in using ACSAT

For the hippocampus dataset A, ACSAT was terminated at iteration n=3 when the change in global threshold value

$$\frac{\left|\tau_{4}^{*}-\tau_{3}^{*}\right|}{\tau_{1}^{*}}=5.12\%<\delta =10\%.$$

For the striatum dataset, ACSAT was terminated at iteration n=1 when the change in global threshold value

$$\frac{\left|\tau_{2}^{*}-\tau_{1}^{*}\right|}{\tau_{1}^{*}}=4.53\%<\delta =10\%.$$

**Figure 7.**
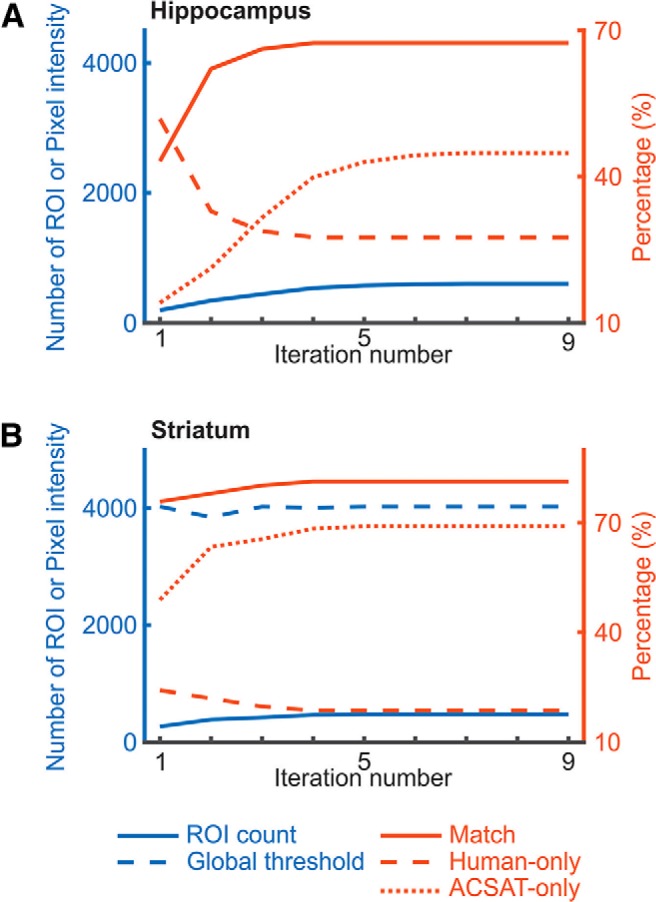
Performance of ACSAT over iterations. ***A***, ***B***, For both hippocampus dataset A (***A***) and striatum dataset (***B***), the cumulative number of identified ROIs (solid line) increased steadily over iterations. The global threshold (dashed blue line) tended to decrease with each iteration, allowing ACSAT to capture ROIs with lower intensity. Both recall (solid red line) and false discovery rate = 1 – precision (dotted red line) increased with iterations, while the false-negative rate (dashed red line) decreased. All results reported here are based on human-generated ROIs before secondary manual inspection of false positives.

To evaluate how ACSAT performs when terminated at different iteration numbers, we ran ACSAT up to nine iterations on both datasets, and calculated several major performance indicators after each iteration (Fig. 7): cumulative number of ROIs, global threshold value, recall, false-negative rate, and false discovery rate (which is equal to 1 – precision) compared to the human-generated ROIs before secondary manual inspection of false positives. The cumulative number of ROIs, recall, and false discovery rate increased with the iteration number, but at different speed. While the cumulative number of ROIs and the false discovery rate increased steadily, recall rose steeply and reached its plateau within approximately three iterations for the hippocampus dataset and after the first iteration for the striatum dataset. Both the global threshold value and the false-negative rate dropped as iterations progressed, indicating that ACSAT dynamically adjusted the threshold to capture potential ROIs with lower intensity in later iterations. This dynamic adjustment of the threshold value at each iteration was possible only because of the removal of segmented ROIs before each iteration. Overall, the changes in these performance indicators over iterations suggested that most true ROIs were identified during the early iterations: n≤3 for the hippocampus dataset and n=1 for the striatum dataset, which are consistent with when the ACSAT termination criterion described by δ was met. ROIs segmented during later iterations were mostly false positive.

#### FIBAT global and local thresholding

**Figure 8.**
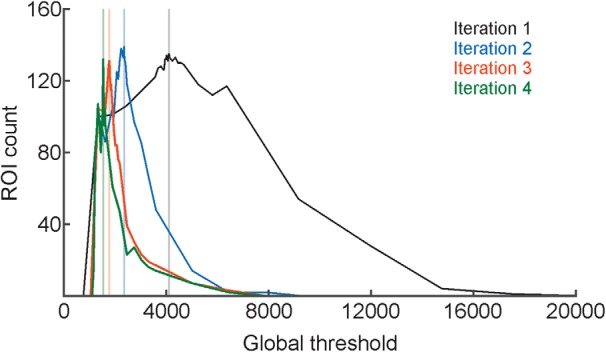
Convergence of the FIBAT optimal global threshold value for the hippocampus dataset A. FIBAT first sampled at a coarse scale across a wide intensity range, and then focused on a small potential intensity range with a fine scale to identify the optimal global threshold value that generated most ROIs. The vertical lines indicate the final optimal global threshold value determined by FIBAT for each iteration.

In Fig. 8, we demonstrate how FIBAT (Fig. 1*B*) determines the threshold value that achieves optimal segmentation results by sampling the distribution of threshold values versus the number of ROIs. Each trace of Fig. 8 plots the number of ROIs that results from each sampled threshold value in the global thresholding step during the first four iterations of ACSAT (Fig. 1*A*) on the hippocampus dataset A. In each iteration, FIBAT (Fig. 1*B*) first samples the threshold values across the entire intensity range at coarse resolution to identify the potential search range that may result in the maximum number of ROIs. FIBAT further resamples threshold values within the new search range with a finer resolution, until it reaches a threshold value that gives the maximum number of ROIs. This design allows FIBAT to determine the optimal threshold value with a fine resolution without actually sampling the whole intensity range at the fine scale, and, as a result, reduces the processing time.

**Figure 9.**
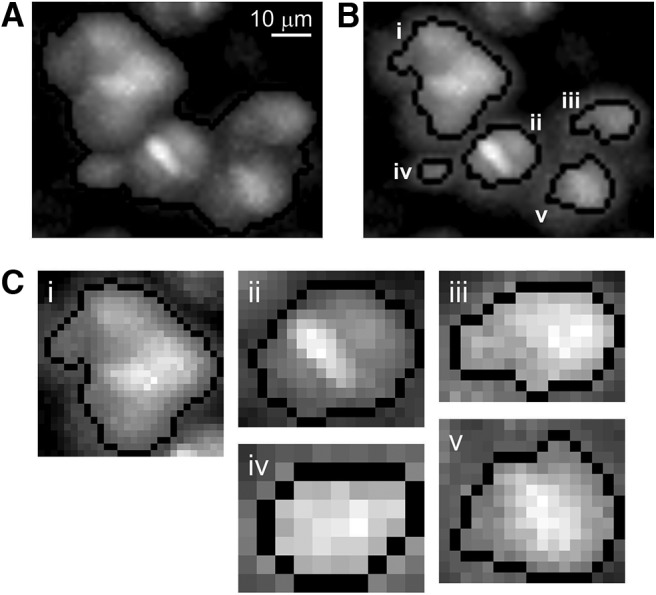
Improved ROI identification by local thresholding. ***A***, With global thresholding alone, a cluster of hippocampal neurons was identified as a single ROI. ***B***, After application of local thresholding, ACSAT successfully separated five new ROIs from the single ROI. ***C***, Zoom-in of each ROI separated by local thresholding.

After performing global thresholding to identify potential ROIs ${\left\{ROIs\right\}}_{n}^{\prime }$ (Fig. 1*A*), ACSAT further applies FIBAT locally to each identified ROI in ${\left\{ROIs\right\}}_{n}^{\prime }$ to refine the segmentation results (Fig. 9). When neurons are densely labeled with GCaMP6, using the global thresholding step alone may lead to one or more large clusters of adjacent neurons being segmented as a single ROI (Fig. 9*A*). For each such cluster, FIBAT (Fig. 1*B*) determines and applies a new threshold value to the local ROI area. With local thresholding, the example cluster is further segmented into five new ROIs (Fig. 9*B*), which would not otherwise be separated by applying the global threshold. Because further local thresholding produces the same result (Fig. 9*C*), the local thresholding step of ACSAT concludes that these five ROIs cannot be further separated, exits the recursive loop, and outputs these ROIs.

### ACSAT performance on two-photon dataset

We applied ACSAT to the two-photon dataset Neurofinder 03.00 (Fig. 11*C*). Genetically Encoded Calcium Indicators are generally not expressed in the nuclei (Tian et al., 2009), and because of the optical sectioning technique that two-photon imaging provides, in this dataset the nuclei appear dark. Additionally, this dataset had high speckle noise. Thus, the time-collapsed image generated by ACSAT using max minus mean pixel values shows bright nuclei. The truth file provided by Neurofinder contains 621 ROIs, most of which are nuclei. Since the features of this dataset are the nuclei, which are smaller, we used the parameters δ=5%, Amin=20 px≈6.9 µm2 and Amax=1000 px≈34.6 µm2 for the global adaptive thresholding step, and Amin=20 px≈6.9 µm2 and $A_{\max}=\infty$ for the local adaptive thresholding step.

ACSAT identified 571 ROIs. Among these, 442 ROIs were matched with the truth (true positive), and 179 ROIs were not in the truth (false positive). Additionally, 129 ROIs in truth were not identified by ACSAT (false negative). This result gave us a recall rate of 71.2% (442 out of 621) and a precision rate of 77.4% (442 out of 571).

We further inspected the time-collapsed image $I_{0}$ and observed that the right side of the time-collapsed image $I_{0}$ had different patterns of texture than the left side. To use the new texture information for ROI detection by ACSAT, we extracted the right side of $I_{0}$ that is rich in texture information to generate ${I^{\prime }}_{0}$ as input to ACSAT. The ${I^{\prime }}_{0}$ was generated by change detection between the original image and its Gaussian-filtered counterpart. Thus, ACSAT identified an additional 157 ROIs, of which 95 were true positives, and 62 were false positives. Combining these additional ROIs with the ROIs identified by direct application of ACSAT results in a recall rate of 82.8% (514 out of 621) and a precision rate of 70.6% (514 out of 728).

### ACSAT performance on cell culture and hippocampus B dataset

Finally, we used ACSAT to detect ROIs in the dataset of the primary neuron culture expressing GCaMP6f (Fig. 11*B*). Qualitatively, it appears ACSAT successfully identified the cell bodies of the majority of neurons in early iterations, and neurites in later iterations. We also used ACSAT to detect ROIs in the hippocampus dataset B (Fig. 11*A*). For both datasets, we used the parameters δ=10%, Amin=50 px≈86 µm2 and Amax=300 px≈516 µm2 for the global adaptive thresholding step, and Amin=20 px≈34 µm2 and $A_{\max}=\infty$ for the local adaptive thresholding step because ROIs tend to shrink in size after repeatedly applying FIBAT.

## Discussion

In this study, we presented our automated cell segmentation by adaptive thresholding (ACSAT) method that adaptively selects threshold values based on image pixel intensity with two iterative steps at the global and local levels using a time-collapsed image. As such, the algorithm is capable of handling morphological variations in fluorescence intensity in neurons and is robust against luminance condition changes across datasets. When applied to two datasets collected from the hippocampus and the striatum in mice, ACSAT resulted in ∼80% recall rate of ROIs containing individual neurons (78.5% for the hippocampus A dataset and 82% for the striatum dataset), and ∼80% precision rate (87% for the hippocampus dataset and 74.1% for the striatum dataset). ACSAT was also able to detect low-intensity ROIs that were initially undetected by human referees. When applied to 500 simulated datasets, ACSAT achieved recall and precision rates higher than 80% when SNR was no less than ∼24 dB. However, the performance of ACSAT falls when SNR reaches below ∼20 dB.

The ACSAT algorithm is an intuitive thresholding method that uses global and local schemes to address variations in fluorescence intensity levels of GCaMP6 fluorescence even within the same image field. Simply applying a lower global threshold value would result in few large ROIs containing multiple neurons within one ROI. On the other hand, with a high global threshold value, only a small number of neurons with high intensity would be found. As such, applying a single high or low threshold value would generate inadequate results of either few or excessive ROIs, which is a universal limitation of thresholding methods. Our algorithm efficiently addresses this challenge in two ways.

**Figure 10.**
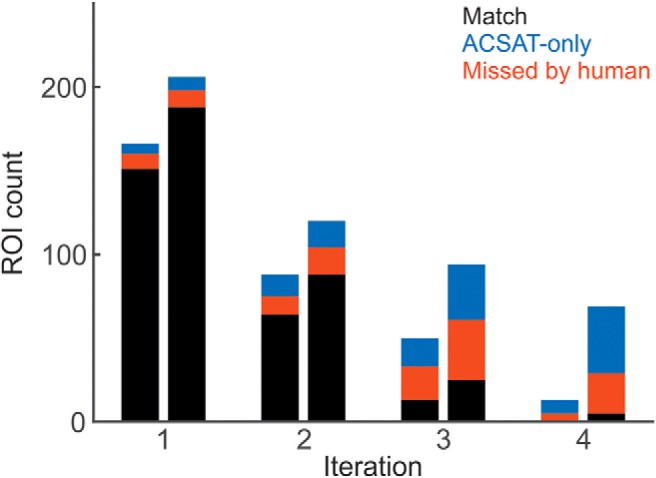
Local thresholding improves ACSAT performance for hippocampus dataset A. The ROIs identified by ACSAT at each iteration before local thresholding (left) and after (right). Local thresholding separated overlapping ROIs and thus helped identify more ROIs, including those identified by human (black) or missed by human (red).

First, it cumulatively excludes previously segmented ROIs from the time-collapsed image $I_{n}$ after each iteration so that in the following iteration, ACSAT could detect new ROIs that require distinct thresholds to separate but were missed with previous thresholds. Therefore, the global threshold value $\tau_{n}^{*}$ (Fig. 1) used by ACSAT usually decreases after each iteration, and ROIs with high intensity were segmented before those with low intensity, as shown in Figs. 3 and 4. Because ACSAT is based on adaptive thresholding, it allows us to objectively and robustly segment ROIs with low intensity relative to the background. These low-intensity areas often pose challenges to human experts when manually detecting ROIs, as our results showed that about half of the ROIs initially labeled as false positive were actually true neurons (Fig. 10).

To evaluate the efficacy of local thresholding, we examined the hippocampus dataset A at each iteration before and after the local thresholding step (Fig. 10, left and right bars, respectively). Local thresholding refined the ROIs detected by global thresholding and captured more true ROIs at every iteration. It is also worth noting that, at later iterations, local thresholding was still able to identify true ROIs that were missed by global thresholding alone (Fig. 10, iteration 4).

**Figure 11.**
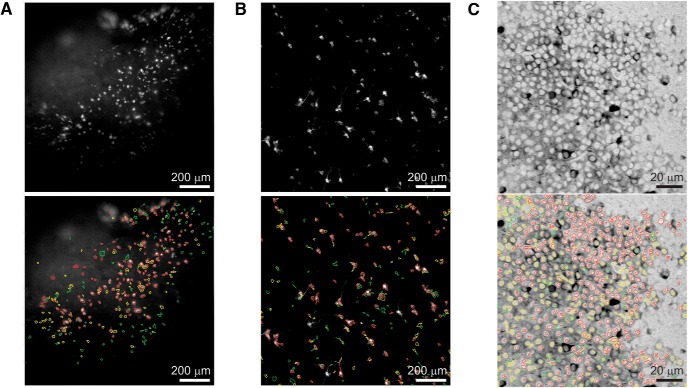
ACSAT results of various datasets. ***A***, The time-collapsed image of hippocampus dataset B (top) with ACSAT ROIs overlaid (bottom). ***B***, The time-collapsed image for the primary neuron culture dataset (top) with ACSAT ROIs overlaid (bottom). ***C***, The time-collapsed image for the two-photon dataset (Neurofinder 03.00; top) with ACSAT ROIs overlaid (bottom). For all three datasets, the majority of ROIs were identified during the first two iterations. Red, yellow, green, and blue ROI outline corresponds to iteration 1, 2, 3, and 4, respectively.

Second, ACSAT uses fluorescence intensity based adaptive thresholding (FIBAT) locally to separate overlapping ROIs. This approach directly addresses the issue of heterogeneity in recorded neural signals when the intensities of pixels surrounding an ROI can vary. However, because a higher thresholding value is usually required to separate adjoining neurons, the output sub-ROIs after local FIBAT are often smaller than the corresponding true neurons. Thus, a simple dilation step was applied during the local FIBAT step. This correction is useful to prevent real ROIs from falling below the minimum area criterion $A_{\min}$ and thus being removed. Although the interleaving process of global FIBAT and local FIBAT has been effective in addressing overlapping neurons, a potential problem still arises if two neurons with similar intensities have significant overlap with each other in the time-collapsed image such that there is no trough between them. Then ACSAT may identify them as a single ROI. Conversely, if there is a neuron with multiple hotspots (Pnevmatikakis et al., 2016), then this may be identified as multiple neurons by ACSAT. Such a scenario, however, can be minimized by the minimum area criterion $A_{\min}$ and the maximum area criterion $A_{\max}$. Spatial overlap is profound for wide-field imaging, but not for two-photon imaging or *in vitro* cell culture imaging with single cell layer. With increasing improvement wide-field imaging, such as volumetric imaging (Shain et al., 2018; Xiao et al., 2018), such significant overlap may be better eliminated during data acquisition step.

ACSAT has three sets of free parameters that can be rationally chosen or otherwise are not sensitive: δ, which describes the termination condition for ACSAT; α, which describes a termination condition for FIBAT; and $A_{\min}$ and $A_{\max}$, which describe the allowed sizes of ROIs.

The termination condition for ACSAT, described by δ, can be explained by the tendencies of ACSAT. Specifically, running ACSAT for more iterations increases the number of ROIs segmented, especially the number of low-intensity ROIs, as the global threshold value $\tau_{n}^{*}$ gradually decreases (Fig. 7). While many of the added ROIs are true ROIs, the proportion of false-positive ROIs added increases as iteration number increases (Fig. 7). This increasing proportion of outputted false positives in later iterations can be attributed to the higher probability of a spurious collection of adjacent background pixels meeting the criteria to be an ROI. Also, the added false positives can be related to the step which clears previously segmented ROIs from the time-collapsed image at the start of each iteration of ACSAT. Due to the scattering of light in brain tissue, ROI removal may leave a few small fragments of bright pixels around removed areas, which could be identified as ROIs during the next iteration. ACSAT tries to avoid this problem by dilating the cleared area, which makes sure the whole ROI is cleared rather than only the brighter center. Besides dilation, these misidentified ROIs were also discarded either because of their small size or because they do not meet the solidity criteria; however, occasionally they may pass the size criteria and become the false-positive ROIs. As a result, the majority of false positives tend to have small size (Fig. 6, yellow).

To balance the effects of simultaneous increase in true ROIs and false positive ROIs, ACSAT stops when a decrease of global threshold value becomes relatively small between iterations, i.e.,

$$\frac{\left|\tau_{n+1}^{*}-\tau_{n}^{*}\right|}{\tau_{1}^{*}}<\delta .$$

At that stage, most true ROIs have been detected and removed from the time-collapsed image. Thus, the global threshold values $\tau_{n}^{*}$ of any further iterations are similar, so most ROIs detected at this stage are false positives. For the hippocampus dataset A, iteration n=3 is when the increase in false positives begins to outweigh the increase in true positives, and for the striatum dataset, nearly all true ROIs segmented by ACSAT were outputted at iteration n=1 (Fig. 7). Qualitatively, the time-collapsed image $I_{0}$ for hippocampus has a higher density of neurons with a greater variety of pixel intensities than the $I_{0}$ for striatum, so it may take more iterations for ACSAT to perform at the same rate on the hippocampus dataset than on the striatum dataset. ACSAT’s performance under the diverse conditions of these two datasets suggests that our choice of δ=10% provides a robust and rational termination condition for ACSAT that can be generalized to other datasets, namely the 500 simulated datasets and the cell culture dataset, as well. In fact, changing the termination condition from δ=10% to δ=5% only affected the segmentation results in <17% of the 500 simulated datasets. For the two-photon dataset, our reported results are using the termination condition δ=5%. In general, users can choose δ to be between 5% and 10% based on the needs of their application: if recall is more important, then users should choose a smaller δ, and if precision is more important, then users should choose a larger δ.

Additionally, the final segmentation results generated by ACSAT are not sensitive to the termination conditions for FIBAT described by α and ε. FIBAT is terminated if the threshold search range has minimal change over an iteration, which we determine in two ways. One way this condition would be satisfied is when all threshold values within the search range result in the same, optimal number of ROIs. This is equivalent to setting the criterion α=100%. For the practical purpose of reducing FIBAT run time, we allow termination if the change in the search range is <1−α=10%. This condition is also easily met when FIBAT is used in the local thresholding step because, by definition, ROIs that cannot be separated by FIBAT will return exactly one ROI no matter what threshold value is used. Additionally, we terminate FIBAT if the search range is smaller than ε, the smallest difference between any pair of adjacent pixels in *I*, which can be objectively and automatically determined from *I*. If FIBAT were to continue refining the threshold value, then the gained precision beyond that defined by ε would be useless due to the discrete step in pixel intensity values in *I*.

The last set of parameters $A_{\min}$ and $A_{\max}$ should be chosen based on how large neurons are expected to be using information including neuron size, image resolution, magnification, imaging method, etc. In our wide-field datasets, the boundaries of neurons may not be as well defined as those collected with two-photon microscope, and the size will appear larger than the size of a neuron due to light scattering in wide-field conditions. This effect is consistent with our observation that the minimum size of the human-generated ROIs was 38 px≈64.6 µm2 for the hippocampus A dataset and 66 px≈112.2 µm2 for the striatum dataset. Thus, our minimum ROI criteria for the wide-field datasets may be larger than a typical neuron size.

The images $I_{0}$ used by ACSAT are time-collapsed, and therefore do not contain any temporal information. With the flexibility of ACSAT, the framework of ACSAT can be used as long as a single image can be generated to represent the ROIs within the image sequence. For example, an input image ${I^{\prime }}_{0}$ can be generated where the value of each pixel represents the time of its maximum intensity. This image ${I^{\prime }}_{0}$ would allow ACSAT to separate adjoined ROIs that have similar intensity values in $I_{0}$ but reach their maximum intensity at different time points, which is described by ${I^{\prime }}_{0}$. Other ways to generate the single representative image include correlations with nearby pixels, intensity dynamics such as standard deviation or variance over time, texture of the time-collapsed image (for example, as used for the two-photon dataset), and a combination of various parameters. Overall, by taking advantage of adaptively determining the threshold value at both the global level and the local level, ACSAT can theoretically perform segmentation on any image containing ROIs with nonhomogenous intensity as long as it has sufficient contrast between ROIs and the background.

10.1523/ENEURO.0056-18.2018.ed2 Extended Data 2 Fluorescence traces in a Matlab struct for all ROIs in the hippocampus dataset A and the striatum dataset grouped into “Match,” “ACSAT,” and “Human” as defined in Fig. 5. Groupings here are based on human-generated ROIs prior to secondary manual inspection of ACSAT-only ROIs. Download Extended Data 2, XLSX file.

## Synthesis

Reviewing Editor: Muriel Thoby-Brisson, CNRS UMR 5287 Université Bordeaux Decisions are customarily a result of the Reviewing Editor and the peer reviewers coming together and discussing their recommendations until a consensus is reached. When revisions are invited, a fact-based synthesis statement explaining their decision and outlining what is needed to prepare a revision will be listed below. The following reviewer(s) agreed to reveal their identity: Nicholas Mellen, Mark Taylor.

Dear Authors,

Your manuscript has been reviewed by two experts and they both agree on the fact that the study is of particular interest for researcher looking for robust methods for the analysis of optical recording data and that the proposed algorithm has merit as a tool in development. However they also pointed out some major concerns that should be addressed. To summarize the main ones: 1) the authors should argue or at least justify most of the analysis parameters and some pre-set values that have been arbitrarily chosen without any explained rationale. 2) Considering the result of human detection analysis as the truth is clearly not correct and cannot be considered as the perfect and only way to test the algorithm performances. 3) The uniformity of the data used to test the algorithm questions the potential performances of the method and the accuracy of the parameters chosen if used on different experimental conditions. The algorithm should be tested on other sets of data acquired in different conditions (calcium indicator, spatial resolution, type of biological sample).

Below are the detailed reviews:

Reviewer 1:

This paper describes a serial thresholding approach to identify ROIs containing groups of neurons, which are then subjected to further thresholding to generate individual ROIs for each neuron in the group.

The challenge of any automated approach to extraction of Ca2+ transients from an image series is to avoid three errors: missing a cell; counting two (or more) cells as one; and counting one cell as two (or more). This problem is analogous to spike sorting from MEA recordings: a plethora of methods have been developed; as yet no single best method has emerged as the consensus “best practice”. This is likely due to at least 2 factors: differences in performance due to MEA design and geometry; and differences due to properties of the tissue being recorded. Further, all spike sorting approaches - and likely all automated Ca2+ transient extraction algorithms - work well on good data (i.e., high S/N, manageable levels of cross-talk between electrodes,...). To differentiate between competing methods, it is more informative to study how algorithm performance degrades as recording conditions deteriorate. Thus it is important to describe the SNR range, and recording conditions over which a given approach is robust.

Comparing algorithms for automated somatic Ca2+ transient detection or spike sorting is complicated by lack of access to ground truth. In the case of optical recording however, it is relatively straight-forward to generate simulation data. A 3D or 4D matrix must be constructed whose values match luminance values of a plane or volume of tissue, with subsets of matrix values (representing somatic Ca2+ transients) varying as a function of time to represent localized, spatially compact somatic Ca2+ dynamics. Both neuronal morphology and neuronal dynamics can be reproduced with sufficient accuracy to render this exercise useful, particularly because Ca2+ dynamics are slower convolved versions of the fast non-linear dynamics of excitable neurons. Benchmarking an algorithm against simulated datasets is essential, because it provides access to ground truth.

Any experimenter using Ca2+ imaging methods is 3 steps removed from this ground truth: under wide-field recording conditions an image series is obtained that collapses activity from a three-dimensional network of neurons onto a plane; Ca2+ transients are extracted from ROIs, which are fitted (with errors) to somata; the resulting time-varying signals are convolutions of the underlying action potentials that give rise to the processing or behavior that is being studied. Simulations are lending rigor to considerations of the limits of what can be inferred from an image series, as a function of indicator Ca2+ binding kinetics, SNR, and underlying neuronal dynamics [1-3]. These papers need to be incorporated into any discussion of automated detection of Ca2+ transients from an image series.

Major concerns:

1. The manuscript does not display any of the Ca2+ traces associated with algorithmically detected ROIs. The authors need to include traces, as well as summary statistics on the signal-to-noise ratio (expressed as deltaF/F or dB) of real positives, false negatives (i.e., cells detected by humans but not by the algorithm), and cells identified by algorithm but not human screeners. Without this information, it is impossible for other researchers to assess whether the methods described in this paper would be suitable for the analysis of their data.

2. The datasets used to validate the algorithm are too similar to one another. Both are generated by in vivo recordings from mice expressing genetically encoded Ca2+ indicators (GECIs). Optical recording from GECIs represents the best case: fluorescence only emanates from neurons expressing the indicator, so static background fluorescence is absent; GECIs bleach less than neurons loaded with synthetic indicator, and are more resilient to phototoxicity, hence recordings can be carried out under more intense illumination. In addition, the spatial resolution of the image series is identical, thus it is impossible to infer how well the parameters selected to analyze these data need to be adjusted to data obtained at higher or lower spatial resolution, and with differing SNR. The authors need to obtain datasets from colleagues, so that the software can be tested on image series obtained from tissue labeled with synthetic indicators, and/or with different spatial resolutions, and/or from slices or tissue culture.

3. Benchmarking the algorithm against “human ground truth” allows us to learn that the algorithm performs slightly less well than a human drawing ROIs on a screen, but this in no way conveys what benchmarking the algorithm against simulations would, where the ground-truth is known, and where algorithm performance can be evaluated as SNR, anatomical distribution, and other features are varied. Use of simulations to accurately benchmark algorithm performance against a known ground truth is a feature of other papers on (semi-)automated approaches to extraction of Ca2+ transients [4-6], and these papers can be consulted to implement simulations that are necessary to accurately benchmark the approach described here.

Minor points

7-8 “With the continued neurotechnology development effort, it is expected that millions of neurons could soon be simultaneously measured.”

There is nothing that I know of in the “neurotechnology development effort” that provides support for the conjecture that millions of neurons will be recorded in parallel any time soon. The authors have ample justification for the development of machine vision methods to analyze optical recordings of brain networks without making extravagant predictions.

11-14“Traditional methods rely mainly on manual or semi-manual inspection, which cannot be scaled to processing large datasets. To address this challenge, we have developed an automated cell segmentation method, which is referred to as Automated Cell Segmentation by Adaptive Thresholding (ACSAT).”

The method described in this paper is not fully automated, but rather semi-automated, since their method generates false positives that must be rejected following inspection. This is generally the case for machine vision approaches to detection of neuronal Ca2+ transients, so in itself it's not a problem. To call the methods presented here as automatic is inaccurate, and should be changed to semi-automated.

16-18” As such, the algorithm is capable of handling morphological variations and dynamic changes in fluorescence intensities in different calcium imaging datasets.”

As indicated in Major Points above, the findings of this paper don't support this broad claim. Only GECI signals recorded at one magnification are analyzed here. To support this claim, the authors need to obtain a broader selection of data to test their methods on. In particular, they need to include data from tissue labeled using synthetic indicator and/or data obtained at higher or lower spatial resolution, and/or data recorded from different types of preparations (slice, tissue culture).

18-20 “In addition, ACSAT computes adaptive threshold values based on a time-collapsed image that is representative of the image sequence, and thus ACSAT provides segmentation results at a fast speed.”

Because of the nature of the problem, analysis of an image series is stupidly parallelizable: either the field of view can be divided up into quadrants, or an image series can be divided up into image subgroups; partitioned data can then be distributed to GPUs for processing. This scalable parallelization reduces the importance of the speed of a particular implementation. Accuracy is more important than speed, because an accurate algorithm can be sped up, but an inaccurate algorithm will be inaccurate regardless of the speed with which it executes.

30-32 “Based on tests performed on two datasets from mouse hippocampus and striatum, ACSAT performed comparable to human referees”

This is inaccurate: the algorithm missed 25% of the cells detected by humans.

64-67 “Principal component analysis (PCA) (Mukamel et al., 2009), as one such approach, requires significant computational resources and CPU processing time, limiting its use in larger datasets”.

Mukamel et al. used ICA not PCA; the second part of the sentence is a non sequitur: the larger the dataset, the more burdensome the computation. Furthermore, Mukamel reduced the computational cost of their approach by applying PCA first to reduce dataset dimensionality. There are other more significant problems (assumption of statistical independence between neurons, requirement of extremely high S/N,...) with Mukamel's approach, but computational efficiency isn't one of them.

68-90 Overview of the literature starting with: “Alternatively, threshold-based methods are simple, intuitive, and fast, and thus are expected to be useful for processing large datasets...”

A waterfall thresholding approach [4] conceptually similar to the methods described here addresses some of the problems described in this survey.

164-168 “To increase time efficiency of the ACSAT algorithm without sacrificing segmentation performance, the inputted image sequence is first collapsed in time into one representative two-dimensional image (ᵃC;0 in Figure 1a), where each pixel in ᵃC;0 is thus represented by the maximum intensity value of that pixel across the entire image sequence with the mean value removed.”

This suggests that the image series is first transformed into a 3D matrix, which is then searched for maximae along the z axis. Add a bit more detail about how the image series is read into matlab.

171 “Because we define a ROI as a non-trivial cluster of adjacent pixels...”

What constitutes a non-trivial cluster? This will vary as a function of spatial resolution determined by magnification, camera pixel size, and binning. Provide more detail.

198-199 “The local adaptive thresholding step (Figure 1c) recursively separates any potentially overlapping ROIs within {ᵄ5;ᵄ2;ᵃC;ᵆ0;}ᵅB; ′ in order to output {ᵄ5;ᵄ2;ᵃC;ᵆ0;}ᵅB;. “

This assumes that troughs in the luminance profiles exist between adjacent neurons; this isn't always the case, particularly if the neurons are stacked along the z-axis. In addition, not all luminance troughs correspond to a boundary between neurons (see {Pnevmatikakis, 2016 #10795}.

215-216 “For the local adaptive thresholding step, we chose ᵃ4;ᵅA;ᵅ6;ᵅB; = 20ᵅD;ᵆ5; ≈ 34ᵰ7;ᵅA;2”

This minimum ROI size exceeds the size of many neurons.

221-222 “A larger ᵄ7; will decrease the probability of skipping the optimal threshold value, but it will result in more computation time that may not be necessary. We chose ᵄ7; = 12.”

The choice of T=12 appears arbitrary. This number is likely too high for recordings at low light levels encoded in the lowest 4 bits of the sensor, and is likely low if light levels are high enough to use the full dynamic range of a 16 bit chip. By working with more heterogeneous datasets, the Authors may arrive at either a heuristic or an algorithmic approach to selecting T.

231-232 “Since ROIs represent real neurons that are roughly spherical in shape and are about 5 μm - 20μm in diameter...”

A 5 um diameter neuron is smaller than A(min).

272-273 “ACSAT is based on adaptive thresholding on a time-collapsed image, and thus it provides segmentation results at very fast speed.”

In the reviewer's experience, generating ROIs isn't the slow step in image processing. The time taken to extract Ca2+ transients is dominated by the number of ROIs and the number of images in the series. No information about total processing time, as opposed to ROI selection is provided. If the authors wish to emphasize speed of computation, they should indicate how processing time to extract Ca2+ transients increases with ROI number.

289-292 “For the hippocampus dataset, ACSAT identified 445 ROIs after three iterations. Among these 445 ROIs, 317 ROIs were matched in the human-generated truth (true positive), and 128 ROIs were not in the human-generated truth (false positive). Additionally, 106 ROIs in human-generated truth were not identified by ACSAT (false negative).”

This passage is difficult to interpret. Why is the label true and false positive assigned to ROIs based on whether they match selections made by humans? A meaningful false positive is generated when the time-varying trace generated by a given ROI shows fluctuations incompatible with neuronal dynamics. If the human and the algorithm agree about an ROI associated with a dead cell, it is nonetheless a false positive.

302-303 “Specifically, for the hippocampus dataset, 70 (54.7%) out of 128 ROIs initially labeled as false positives were later determined to be actual 304 neurons,”

Determined how? Based on what? Show the traces (ideally all the traces in supplemental materials, and a representative sampling in a figure in the main text), show summary statistics of S/N for each of these groups.

As an aside, going through traces from algorithmically generated ROIs is exactly the step that puts these methods in the “semi-automated” category.

329-334 “To evaluate how ACSAT performs when terminated at different iteration numbers, we ran ACSAT up to 9 iterations on both datasets, and calculated several major performance indicators after each iteration (Figure 5): cumulative number of ROIs, global threshold value, recall, false negative rate, and false discovery rate (which is equal to 1- precision) compared to the human-generated truth prior to secondary manual inspection of false positives”

Missing in this manuscript are general methods for experimenters to select appropriate iteration numbers when they don't have the results of analysis by humans to benchmark against. Robust simple methods for applying these methods when no alternative estimate of “ground truth” is available is essential, because these are the conditions under which other researchers will make use of these algorithms.

413-415“Because ROIs generally have pixel intensity decreasing radially, this shrinking effect is expected to be uniform around an ROI and thus can be corrected by a simple dilation”

If this operation is performed, it should be included in Methods. Further, Mukamel showed that smaller ROIs fitted to the brightest region of somatic Ca2+ transients improved SNR, thus expanding the ROIs may be counterproductive.

Overall, the discussion is more an overview of the heuristics used by the authors to obtain optimal performance from their processing approach. Missing entirely from the discussion is a critical evaluation of the software, and ideally a description of conditions under which their approach would produce sub-optimal segmentation. This should be added to the discussion.

1. Lutcke, H., et al., Inference of neuronal network spike dynamics and topology from calcium imaging data. Front Neural Circuits, 2013. 7: p. 201.

2. Hamel, E.J., et al., Cellular level brain imaging in behaving mammals: an engineering approach. Neuron, 2015. 86(1): p. 140-59.

3. Wilt, B.A., J.E. Fitzgerald, and M.J. Schnitzer, Photon shot noise limits on optical detection of neuronal spikes and estimation of spike timing. Biophys J, 2013. 104(1): p. 51-62.

4. Mellen, N.M. and C.M. Tuong, Semi-automated region of interest generation for the analysis of optically recorded neuronal activity. Neuroimage, 2009. 47(4): p. 1331-40.

5. Mukamel, E.A., A. Nimmerjahn, and M.J. Schnitzer, Automated analysis of cellular signals from large-scale calcium imaging data. Neuron, 2009. 63(6): p. 747-60.

6. Pnevmatikakis, E.A., et al., Simultaneous Denoising, Deconvolution, and Demixing of Calcium Imaging Data. Neuron, 2016. 89(2): p. 285-99.

Reviewer 2:

Overall, this work provides a timely and welcome attempt at automated cell segmentation analyze through new iterative global and local thresholding approaches. The approach and findings have considerable merit for neurobiology applications and future advances in the speed and accuracy of in situ data analysis. There are several issues to address. Comments and questions are offered below.

1) One caveat to the algorithm being performed on time-collapsed, max-intensity projected data is that transient data, particularly brief events embedded within a long sample period, would be diluted and likely overlooked. Moreover, noise as well as signal accumulates with this method, promoting false-positives.

2) The problem with the concept of “human truth” is that, by the authors' own demonstration, such a thing doesn't exist. Comparing the algorithm performance to a separate but clearly fallible human analysis doesn't necessarily test the algorithm. This is reinforced by the process undertaken here to re-evaluate false-positive data which were subsequently “confirmed” to be cells. Assuming this was done by further visual inspection, the gold standard simply becomes iterative human inspection and not truth. There is no issue with comparing to human evaluations but it should not be considered truth. A computer-generated data set should be included to assess the algorithm, wherein regions of different (but known) size and intensity are placed within comparable backgrounds.

One potential additional problem of iterative/thresholding segmentation is that cells with multiple hot-spots might be fragmented as separate ROI's (e.g. see Fig 7b, ROI's ii and iv?) and adjacent cells having similar activity may remain unsegmented (e.g. see Fig 7b, ROI i?). This could be a problem if the intent is to identify individual cells rather than active sites. One way to determine whether ROI's represent cells (particularly, the residual small segments from iterative analysis) is to test the algorithm on cells or excised tissue that has been counterstained with a nuclear dye. Additional problems may result from dilation of removed ROI's, as this could create new detection problems with iterative analysis. This should be discussed.

3) Pg 8, The statement, “...non-trivial cluster of adjacent pixels with high intensity values...” is vague. How many adjacent pixels at what intensity?

4) A number of analysis parameters/pre-set values were “chosen” by the authors but little perspective is provided for why. The authors should provide some rationale for non-arbitrary criteria. Why was delta set to 10%. A sweep of different cut-off levels might reveal the optimal value.

5) It is not clear what is meant by “clearing or removing previous ROI's” before the next iteration. Are the pixels literally removed, considered 0, other?

Other comments:

Pg 6, Headfixed or head-fixed

Fonts of 'Figure' references in text are inconsistent with document.

As indicated above, the terms 'human truth' and 'human-generated truth' are not meaningful or particularly useful.
